# Significant effect of image contrast enhancement on weld defect detection

**DOI:** 10.1371/journal.pone.0306010

**Published:** 2024-06-28

**Authors:** Wan Azani Mustafa, Haniza Yazid, Hiam Alquran, Yazan Al-Issa, Syahrul Junaini

**Affiliations:** 1 Advanced Computing (AdvCOMP), Centre of Excellence, Universiti Malaysia Perlis (UniMAP), Pauh Putra Campus, Arau, Perlis, Malaysia; 2 Faculty of Electrical Engineering Technology, Universiti Malaysia Perlis (UniMAP), Pauh Putra Campus, Arau, Perlis, Malaysia; 3 Faculty of Electronic Engineering Technology, Universiti Malaysia Perlis (UniMAP), Pauh Putra Campus, Arau, Perlis, Malaysia; 4 Department of Biomedical Systems and Informatics Engineering, Yarmouk University, Irbid, Jordan; 5 Department of Computer Engineering, Yarmouk University, Irbid, Jordan; 6 Faculty of Computer Science & Information Technology, Universiti Malaysia Sarawak, Kota Samarahan, Sarawak, Malaysia; Southwest Petroleum University, CHINA

## Abstract

Weld defect inspection is an essential aspect of testing in industries field. From a human viewpoint, a manual inspection can make appropriate justification more difficult and lead to incorrect identification during weld defect detection. Weld defect inspection uses X-radiography testing, which is now mostly outdated. Recently, numerous researchers have utilized X-radiography digital images to inspect the defect. As a result, for error-free inspection, an autonomous weld detection and classification system are required. One of the most difficult issues in the field of image processing, particularly for enhancing image quality, is the issue of contrast variation and luminosity. Enhancement is carried out by adjusting the brightness of the dark or bright intensity to boost segmentation performance and image quality. To equalize contrast variation and luminosity, many different approaches have recently been put forth. In this research, a novel approach called Hybrid Statistical Enhancement (HSE), which is based on a direct strategy using statistical data, is proposed. The HSE method divided each pixel into three groups, the foreground, border, and problematic region, using the mean and standard deviation of a global and local neighborhood (luminosity and contrast). To illustrate the impact of the HSE method on the segmentation or detection stage, the datasets, specifically the weld defect image, were used. Bernsen and Otsu’s methods are the two segmentation techniques utilized. The findings from the objective and visual elements demonstrated that the HSE approach might automatically improve segmentation output while effectively enhancing contrast variation and normalizing luminosity. In comparison to the Homomorphic Filter (HF) and Difference of Gaussian (DoG) approaches, the segmentation results for HSE images had the lowest result according to Misclassification Error (ME). After being applied to the HSE images during the segmentation stage, every quantitative result showed an increase. For example, accuracy increased from 64.171 to 84.964. In summary, the application of the HSE method has resulted in an effective and efficient outcome for background correction as well as improving the quality of images.

## Introduction

Many researchers agreed that pre-processing is an essential stage in regard to image analysis [1, 2]. Contrast variation and luminosity problems are commonly affected by occlusion, pose, and lighting, causing difficulties in the segmentation process [3–6]. Contrast and luminosity enhancement are important, where it cannot build a perfect mathematical model, in particular with respect to extreme illumination [7]. Usually, the researcher proposes various approaches to eliminate uneven illumination in an image. However, the proposed methods are unsuccessful if the images have both luminosity and contrast problems [7, 8]. Many enhancement methods were established in the past few decades for specific types of images and applications based on the literature. Still, there is no single method applicable to solve the contrast variation and luminosity simultaneously. This served as the driving force behind the current investigation. As per earlier studies, the key challenge is identifying a dividing line to distinguish between the bright and the dark area prior to using the contrast enhancement method. Secondly, the cut-off value is the primary issue when thinking about filtering methods (for instance, homomorphic filtering). The literature states that the researchers manually tested the cut-off and other factors to acquire them [9, 10]. For all varieties of non-uniform images, the parameter value is inefficient and inaccurate. To the greatest of the author’s knowledge, several instances in the literature systematically detail the influence of contrast variation prior to the segmentation process, even though many review analyses focused on contrast enhancement [11–13]. In the segmentation process, the contrast and illumination effect are important since the non-uniform contrast images will reduce the effectiveness of the segmentation result. According to the study by [14], the image with uneven illumination and contrast variability significantly affect the vertebral bone segmentation process. This paper presented a comprehensive review of three contrast enhancement techniques, namely histogram equalisation (HE), gamma correction (GC), contrast limited adaptive histogram equalisation (CLAHE), as well as the effect on the segmentation performance. A research finding by [13] also points to non-uniform skin image’s impact on the segmentation accuracy. Similarly, low contrast is important to be solved before applying the segmentation process. The enhancement methods were proposed to normalise the low contrast effect and automatically improve the image quality [15–17].

## Literature review

Weld defect inspection from radiography films is essential for ensuring weld joint’s serviceability and safety. Due to the human interpretation’s limitations, the establishment of novel computer-aided algorithms with respect to automated detection coming from radiography images has become a focus of current research. Pre-processing, defect classification, and defect segmentation is three parts of automated defect inspection. First, the classic defect classification approach based on feature selection, extraction, as well as the classifier is presented in terms of its accomplishments and limits. The applications of innovative learning-based models (particularly deep learning) were then discussed [18].

In 2018, Kalaiselvi and John Aravindhar [19] developed a computer-aided detection (CAD) system depending on image processing techniques to identify weld defects. Here, X-ray images are used in non-destructive testing. Gradient image creation, filtration using the Gaussian pyramidal filters technique, and segmentation employing the Expectation and Maximization (EM) algorithm are the three phases of the suggested system. The suggested system’s performance is evaluated by comparing the segmented image’s sensitivity, specificity, as well as accuracy to its associated ground truth images. With the same goal in mind, Wang et al. [20] released a study outlining an integrative strategy based on magneto-optical imaging (MOI) that joints novel image capture, filtering, as well as enhancement algorithms for orthogonal weld defect detection. A variety of images were obtained, but only a select handful was employed for further processing using the conventional method, which relies on human judgment by tossing out images with defects. To balance the image intensity, the normalization approach is performed, followed by edge extraction and image fusion utilizing a 2D gradient method. Yan et al. [21] also looked at the radiographic images at a variety of intensities and scales. To ensure that no defects are missed, a multi-scale, multi-intensity parameter space is first generated, and pre-processed images that meet the parameters are then employed. The pre-processed image is optimized based on the weld detection standard and the characteristics of the radiography image, after which the parameter value range is automatically limited. To reduce false detections and precisely portray defect boundaries, algorithms for integrating as well as screening the defects in various pre-processed images are created. The method suggested in this research is ubiquitous, resilient, and accurate, according to experimental data. They then suggested building 3D-depth as well as 2D-gray imaging of the bead surface to detect typical surface defects in aluminium alloy weld beads [22]. In this vision system, structured laser light is used to obtain the bead surface’s 3D-depth image, and multi-angle illuminations are used to obtain grayscale images. Then, four methods are offered for extracting the weld bead boundaries based on the distinctive features visible in the 3D depth and 2D grey images. Defects including undercut, collapse, burning-through, surface porosity, excessive reinforcing, spatter as well as poor forming are analyzed using the 3D depth image.

The data is frequently imbalanced when a sensor data-based detection approach is used to look for potential defects in industrial products. Moreover, this issue has a negative impact on the defect detection system’s resilience and accuracy. The following is an example of a welding defect detection method depending on imbalanced radiographic images. To address the data imbalance as well as enhance the consistency of defect detection, Guo et al. [23] studied a welding defect detection technique that combines transfer learning and a generative adversarial network. It is first recommended to use a unique model called a contrast enhancement conditional generative adversarial network as a creative global resampling strategy for enhancing X-ray image data. The data distribution in the image is balanced, and the number of image samples is raised while dealing with the issue of how feature extraction is limited by weak contrast in some images. Weld defects, for instance, porosity, tungsten inclusions, gas pores, longitudinal cracks, slag inclusions, and lack of penetration, are considered by Malarvel & Singh [24]. They recommended using a multi-class support vector machine (MSVM) in X-radiography images as an autonomous method for weld defects detection and classification. This proposed technique consists of two parts. First, the images were smoothed utilizing a modified anisotropic diffusion approach in the first module. Then, in the second module, the segmentation procedure was carried out employing an upgraded Otsu’s approach. Lastly, the characteristics of the region of interest are retrieved and fed into a multi-class support vector machine that utilizes the kernel Gaussian radial basis function as an input. The proposed method was compared against Bayes, multi-layer perceptrons, and MSVM with polynomial kernel functions, as well as artificial neural networks (ANN). Based on the implementation and testing findings, the recommended method can successfully identify and classify weld defects in X-radiography images. The non-zero pixel approach, on the other hand, was suggested by Zhang et al. [25] to quantitatively analyze and display deep learning features. The CNN model has fully utilized the arc lights by integrating them in a variety of ways to provide complementary features, as opposed to eliminating arc light interference as is typically done. With a 99.38% mean classification accuracy, the CNN model outperforms. This study may be used to assist the quality control in real-time laser welding and metal additive manufacturing (AM).

This paper’s primary goal is to examine how applying contrast enhancement to weld defect images prior to binarization affects the ability to detect those defects. This study concentrates on the post-processing stage, and the binarization of the corrected images produced some striking results. With the other illumination options, the contrast variation issue is successfully reduced by this pre-processing procedure. Last but not least, this discovery can help numerous studies focus on the binarization technique or post-processing stage. In addition, a few assessment techniques and segmentation methods were explained for measurement analysis and comparison purposes. In the segmentation stage, the F-Measure, Peak Signal Noise Ratio (PSNR), and Accuracy were obtained to assess the performance. The remaining sections of the paper are arranged as follows. While Section 2 discusses literature from earlier studies, Section 1.1 offers the relevant introduction. Additionally, Section 3 gives the suggested method of augmentation and segmentation, and Section 4 displays the results of the experiment and a comparison with several chosen approaches. Finally, Section 5 brings this study to a conclusion.

## Methods

Contrast enhancement and luminosity are important stages in pre-processing images to improve their quality for specific applications, which can be used in human vision or for further machine processing. The term "contrast" often refers to the distinction or relationship between the intensity of a certain feature and its surroundings [26]. Meanwhile, [27] defined contrast as the relative difference between a central object and a surrounding region of the given pixel. The bad contrast images commonly cause noise and blurring. Luminosity refers to the spot of brightness that appears in a certain region on the original image. The intensity of the luminosity is greater in comparison to the foreground and background. The main cause of the luminosity is the lighting problem [28].

**Fig 1:**
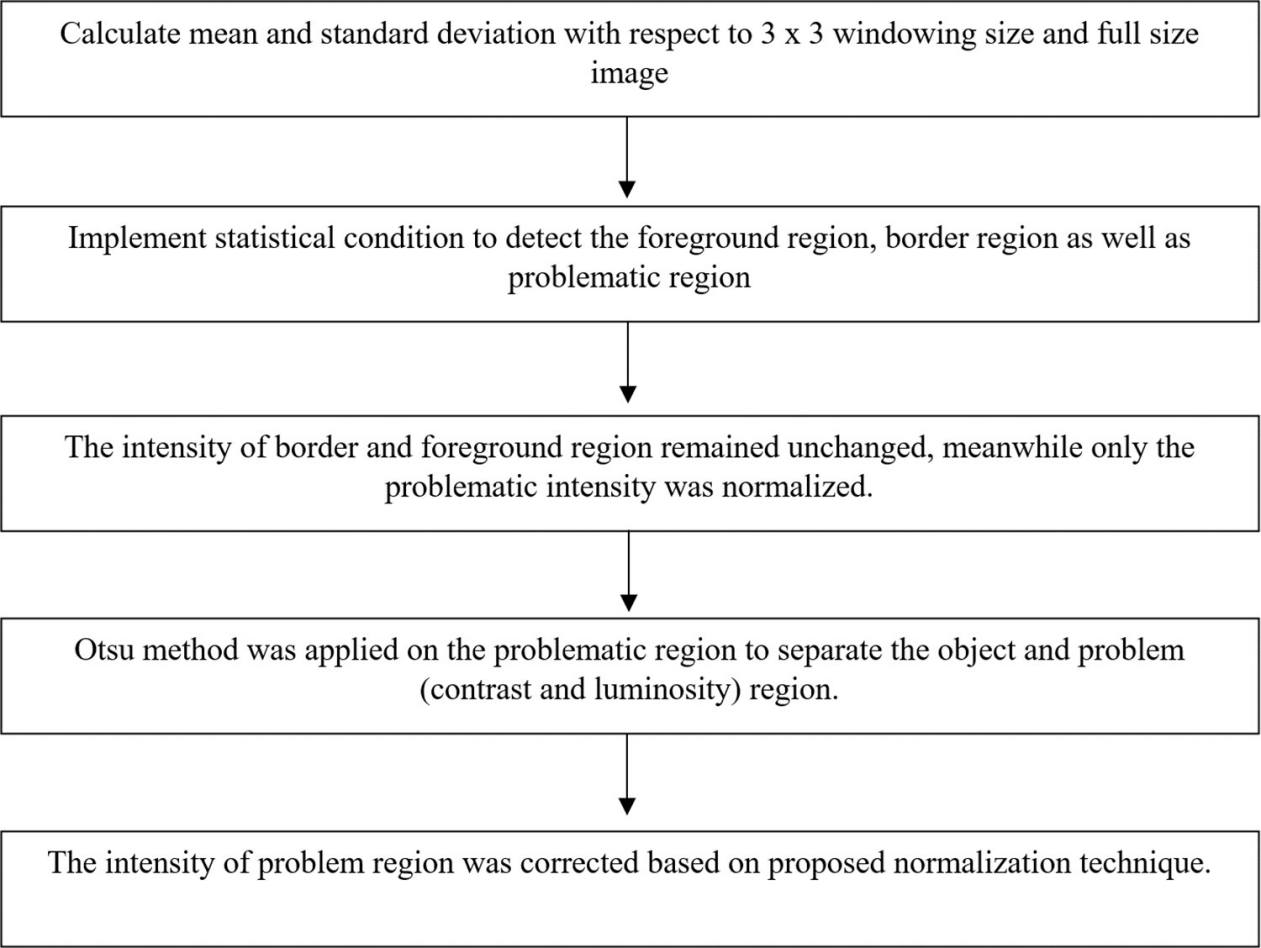
Steps of the proposed HSE method [29].

**Fig 2:**
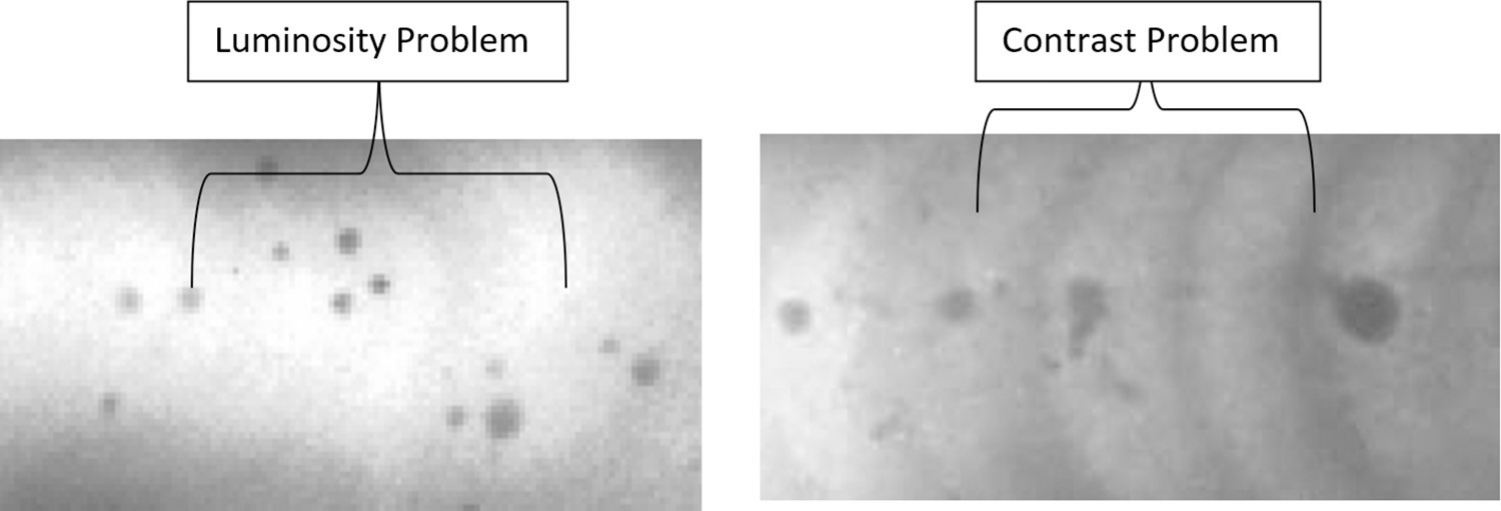
Weld defect images after capture by SEM.

The pre-processing stages focus on the problematic region in order to normalize the contrast and luminosity problem. Based on Fig 1, after detecting the problematic region, the original image undergoes two normalization stages; the first level and the second level. Each normalization stage involved two important parts: (1) the threshold value to separate the problematic and non-problematic regions, and (2) the normalized intensity was obtained to replace the problematic intensity. At the same time, the original intensity of the foreground and border remained unchanged. This is the important key to remaining the details of the original information. Finally, the three regions (foreground, border, and problematic) were combined to create the final corrected image. In conclusion, the steps of the proposed method can be summarized in Fig 1. Otherswise, Fig 2 show the problem of luminosity and contrast on weld defect images.

### Otsu method

Based on both between-class and global variance, the approach automatically determined the threshold value. To propose the final technique, Otsu assumes that the non-uniform image has two areas: dark and bright [30]. Lastly, Otsu thresholding is based on:

$$k=\frac{\sigma^{2}B}{\sigma^{2}G},$$ (1)

in which *k* denotes a threshold value, *σ*^2^*B* represents a global variance of the entire image, whereas *σ*^2^*G* resembles between-class variance.

#### Bernsen method

In order to calculate a local threshold value for each pixel, the Bernsen algorithm is based on estimation. When the difference between the minimum and maximum grey level value exceeds a threshold *k*, this number is solely designated as the local threshold value. If not, it is presumed that the window region only has pixels from one class (background or foreground). The default windowing size (*w*) is 3 x 3 and *k* is 15 [31]. The following is the final equation:

$$T\left(x,y\right)=\frac{Z_{\max}+Z_{\min}}{2},$$ (2)

in which *Z*_*min*_ and *Z*_*max*_ represent the lowest and highest grey level pixel values.

#### Image quality assessments

The objective as well as subjective methods are both used to assess image quality. Since several observers must be chosen and rate the images’ quality according to their personal judgments, the subjective method evaluation is regarded as expensive, time-consuming, and labor-intensive [32]. Without human intervention, the objective evaluation evaluates the image’s quality using automatic algorithms [33]. A number of limited objective evaluations are used in the current research project.

*1*. *Peak Signal Noise Ratio (PSNR)*. Peak signal-to-noise ratio (PSNR), also known as the ratio between the maximum intensity of an image and the noise that distorts it and influences the accuracy of its representation, is a technical term. The logarithmic decibel scale is typically used to express PSNR. A higher PSNR value will result in a restored image with better visual quality [34]. The equation for the PSNR also involved the Mean Square Error (MSE) which is represents the cumulative squared error between the compressed and the original image and final calculation as follows:

$$PSNR=10\log_{10}\frac{(2^{n}-1{)}^{2}}{\sqrt{MSE}}.$$ (3)

*2*. *Misclassification Error (ME)*. Misclassification is described as a variable for interpretation, analysis, and, if the misclassification is disregarded, resulting in biassed estimation. The following equation and method performance are assessed using the misclassification error (ME):

$$ME=1-\frac{|B_{0}\cap B_{T}|+|F_{0}\cap F_{T}|}{|B_{0}|+|F_{0}|}$$ (4)

in which *B*_0_ and *F*_0_ represent the background and foreground of the original image, whereas *B*_*T*_ and *F*_*T*_ represents the background and foreground of the test image [35]. The ME displays the percentages of background pixels that are mistakenly designated as foreground, and vice versa, the background pixels that are incorrectly designated as forefront. This might range from 0 for an ideal classified image to 1 for a completely incorrect binarized image.

*3*. *F-Measure*. F-score compromises the foreground and background pixels. It represents the direct relationship between sensitivity and precision [36]. The corresponding equation reveals its relation:

$$F-measure=\frac{2.Precsion\times recall}{Precision+recall}$$ (5)

*4*. *Accuracy*. It indicates the prediction results of objects. The equation is represented as follows:

$$Accuracy=\frac{TP+TN}{TP+TN+FN+FP},$$ (6)

In which TN denotes True Negative, TP represents True Positive, FP refers to False Positive, whereas FN is False Negative. Its value ranges from [0, 1]. As it gets close to 1, the segmentation is better [37].

## Results

**Fig 3:**
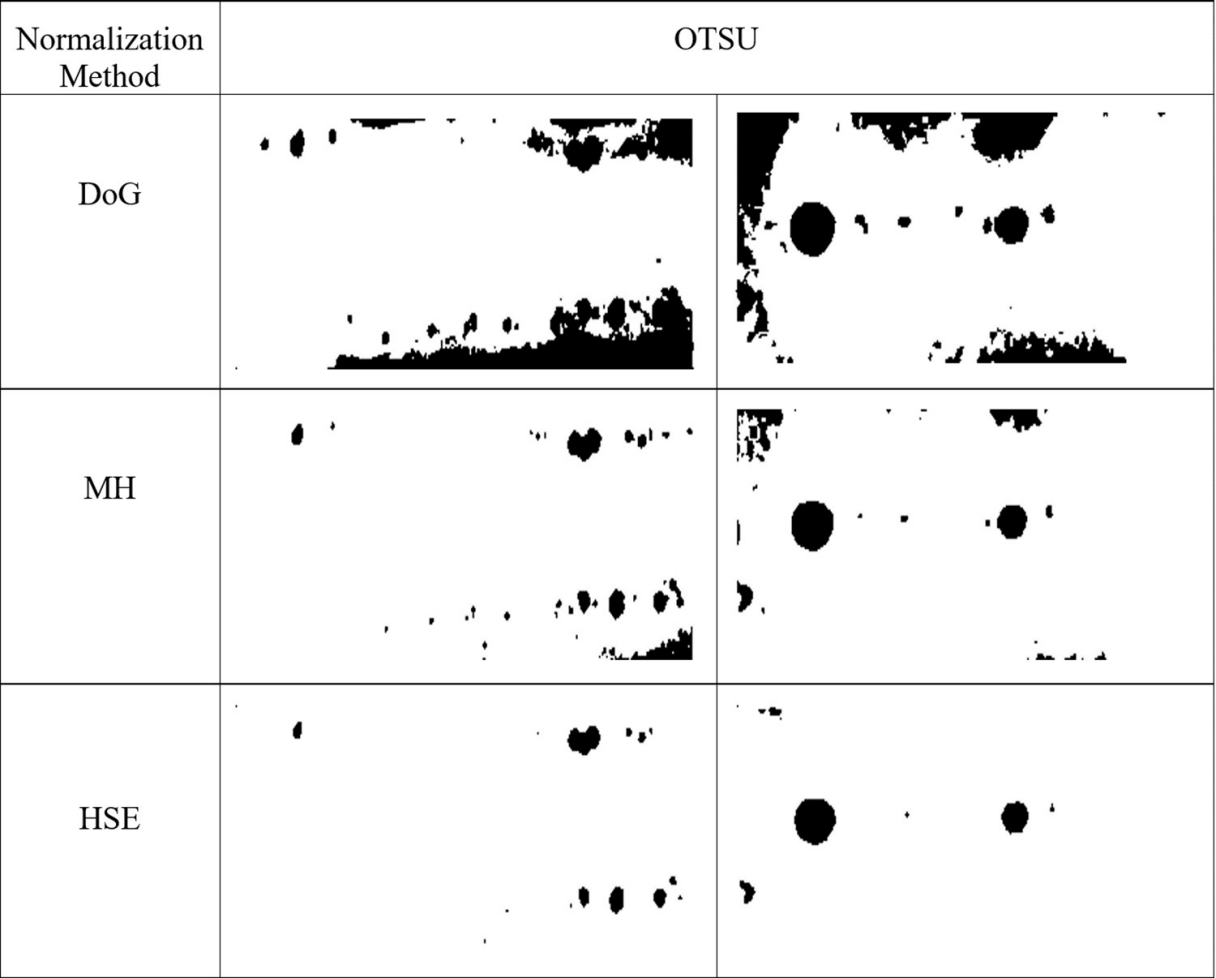
Weld defective images using Otsu segmentation with various normalization methods.

The contrast enhancement plays a fundamental role in order to improve the segmentation result. Normally, a good contrast image automatically produces better segmentation [13, 28]. In this research, to verify the effectiveness and efficiency of normalization methods, the segmentation based on Otsu Thresholding was applied. Fig 3 shows the comparison of resulting images from different normalization methods after using the Otsu method. According to Fig 3, the results of defect segmentation using the enhanced image is better and comparable to the benchmark. However, the DoG and MH methods produced some artifacts in the segmentation result. From the observation, the HSE method can produce the correct image in order to increase the segmentation result. In contrast, other methods are unsuccessful in enhancing the image properly and significantly fail in segmenting the object correctly.

As presented in Fig 3, the HSE method is able to improve the contrast of problematic regions, which leads to better segmentation results. On the other hand, DoG and MH enhanced the contrast of all images, not only a specific region.

**Fig 4:**
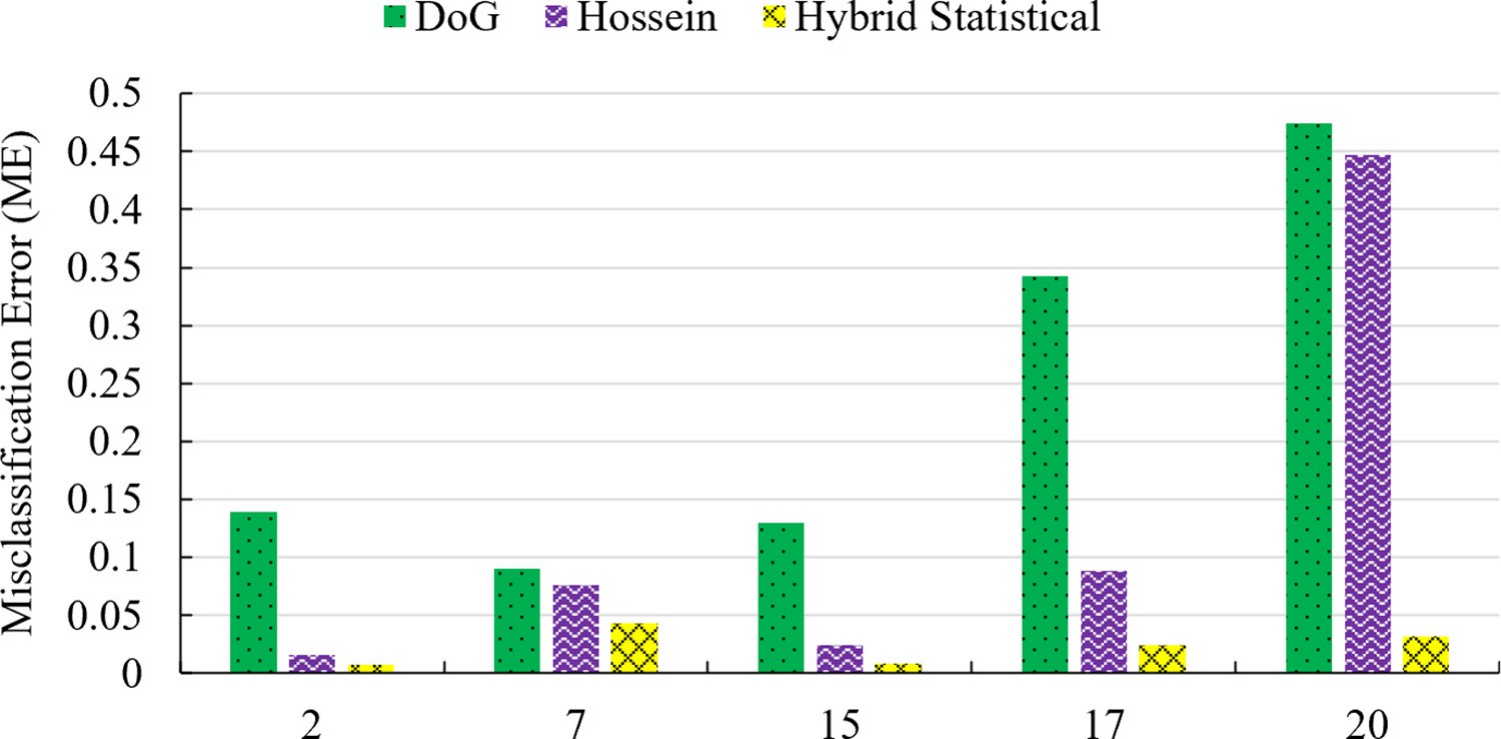
The comparison results are based on ME for the weld defect dataset.

Therefore, poor segmentation results leads are appeared after applying Otsu thresholding. Next, the ME is calculated to quantitatively assess the segmentation result’s quality. Five images were selected for comparison purposes, and the result is shown in Fig 4. Again, a lower ME value suggests that the segmentation result is of higher quality. Here, the x-axis expresses the number of images that have been used, and the y-axis shows the mean error for each method with a different number of images. The figure compares the misclassification error rates of three methods (DoG, Hossein, and Hybrid Statistical) applied to a weld defect dataset across varying numbers of images (2, 7, 15, 17, and 20). The DoG method consistently exhibits the lowest misclassification error rate across all image counts, indicating superior performance over the other two methods for this dataset. At lower image counts (2 and 7), Hossein has a slightly lower error rate than Hybrid Statistical, but as the number of images increases (15, 17, and 20), both Hossein and Hybrid Statistical have comparable and higher error rates than DoG. The Hybrid Statistical method generally has the highest misclassification error rate, especially at the highest image count of 20.

According to Fig 4, the average of the five images produced by the HSE approach is the smallest when contrasted with the other methods for various types of images. However, the enhanced image from the HSE approach significantly produces a big improvement in image segmentation as the number of images grew compared to the DoG and MH methods.

Then, to make quantitative analyses of the HSE method, a comparison based on segmentation was implemented. Comparing the performance of the results between the original image and the enhanced image is the goal of image segmentation. Typically, the defect dataset was proposed for segmentation utilizing the Otsu and Bernsen methods [38]. In this research, the segmentation was demonstrated to verify the effectiveness of the enhanced image from the HSE method. The performance after applying HSE before segmentation is clear when compared with other methods like MH and DoG. Three segmentation evaluations, namely F-measure, PSNR, and Accuracy, were determined in order to check the method’s efficacy. Table 1 displays the quantitative assessment results’ average.

**Table 1:** The quality assessment of the segmentation result.

Segmentation Method	F-Measure (%)		PSNR		Accuracy (%)	
	Original	HSE	Original	HSE	Original	HSE
**Otsu**	20.2083	41.9133	5.0739	10.6310	64.1711	84.9644
**Bernsen**	22.7352	23.9717	5.5116	5.8439	60.9076	65.0791

Based on Table 1, the segmentation result of the enhanced image clearly improves compared to the original image, either for the Otsu and Bernsen methods. The F-measure shows the increment from 20% to 41% (Otsu method) and 22% to 23% (Bernsen method). Overall, the Bernsen method shows the smallest improvement compared to the Otsu method. Finally, to show the contribution of the HSE method, the percentage increments for the three assessment techniques are obtained. The Otsu method shows the biggest average improvement, which is 82.67%, while the Bernsen method is 6%. In summary, the HSE method is successful and effective in increasing the segmentation performance.

**Fig 5:**
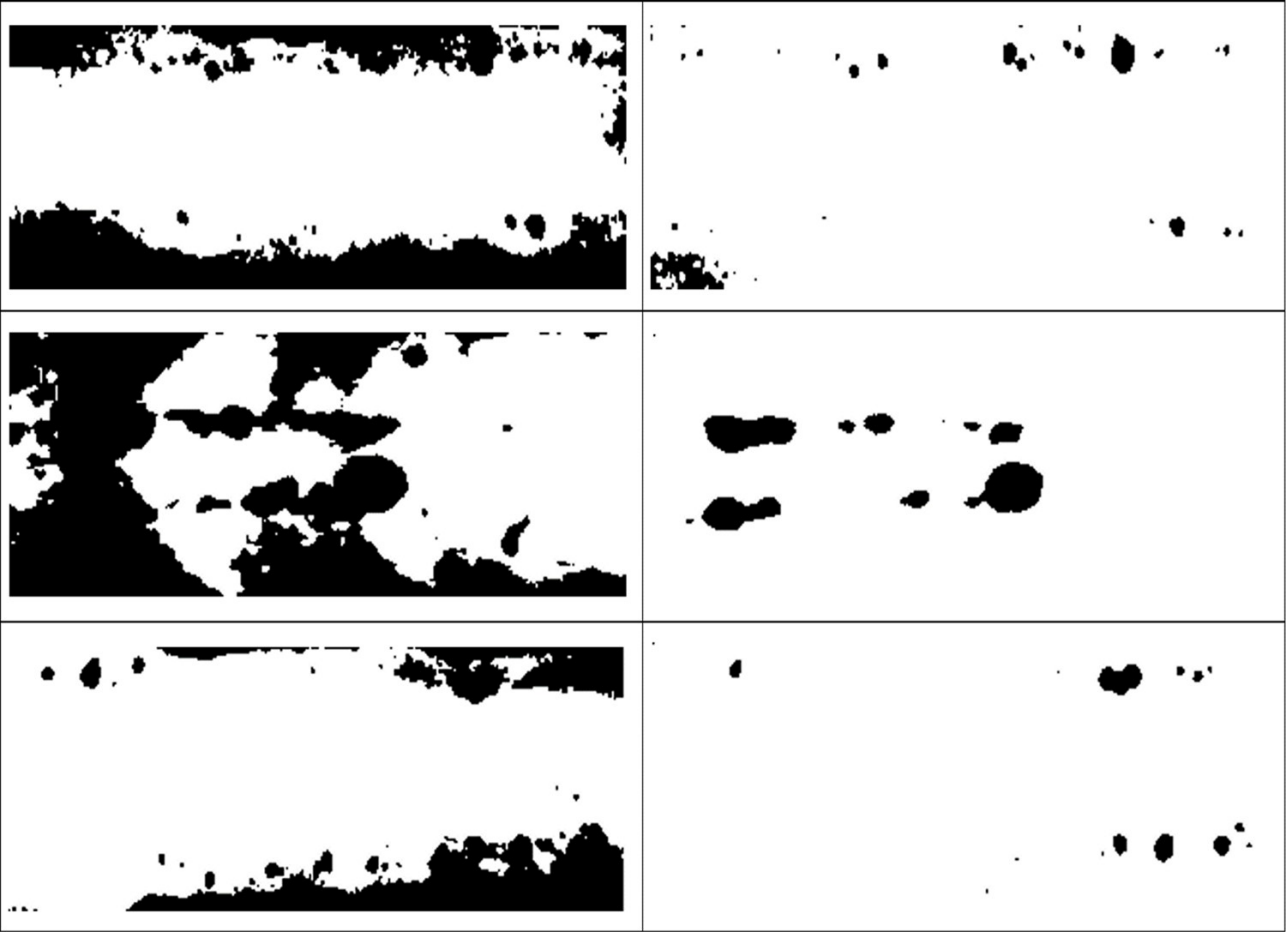
The resulting image after applying Otsu segmentation. (a) original image and (b) HSE image.

**Fig 6:**
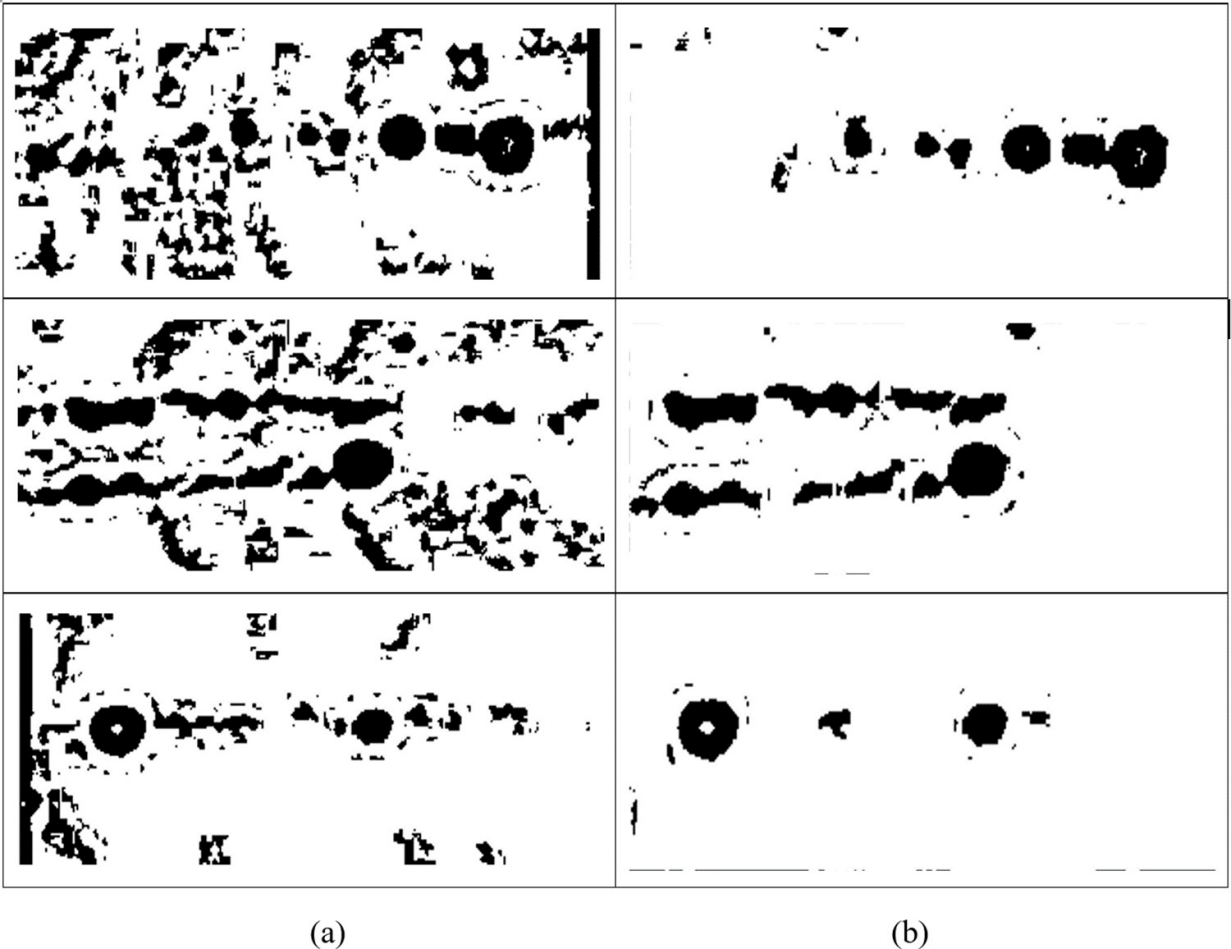
The resulting image after applying the Bernsen method. (a) original image and (b) HSE image.

The first experiment was performed in order to compare the HSE method’s performance based on Otsu segmentation, as shown in Fig 5. Based on the observation, the result of the Otsu method clearly shows an improvement compared to the original image. However, the original images look degraded with artifacts. Second, the Bernsen method was tested on the enhanced image. The resulting image using the Bernsen method is presented in Fig 6. Based on Fig 6, the observations demonstrate that the HSE method was able to improve the Bernsen segmentation result as well.

## Conclusion and future work

In image processing, poor illumination affects the quality of the image, particularly the contrast between the bright and dark regions. Reviews of the literature have shown how the segmentation process is impacted by variations in contrast and luminosity. Despite the fact that numerous studies had presented an enhancement strategy, they had only considered contrast variation enhancement. Therefore, a fresh approach that can be used for both luminosity and contrast issues must be suggested. This work proposes an improved way to normalize the brightness and contrast variation problem based on a mix of direct methodology and statistical data. The suggested method divided the local and global neighborhoods into three groups based on mean and standard deviation: the foreground, border, as well as problematic region (luminosity and contrast).

Enhancing the luminosity and contrast of weld defect images is crucial for improving the visibility and clarity of defects, enabling more accurate identification and measurement. This enhancement highlights fine details, improves edge definition, and distinguishes between different phases or materials within the weld, facilitating both manual and automated analysis. Clearer images support efficient quality control, comply with industry standards, and provide valuable documentation for technical reports and research publications. Additionally, they aid in research and development by offering detailed insights into weld microstructures, and serve as effective educational tools for training professionals and students in weld defect detection and analysis.

In this paper, the HSE method was proposed depending on statistical parameters such as mean and standard deviation. The primary goal is to automatically improve the segmentation result and normalize the problematic region (contrast & luminosity). The original region in this study was divided into three groups: the foreground, the border, as well as the problematic region. A new normalization intensity was used to substitute the contrast as well as luminosity region, whereas the foreground and background intensity remained the original intensity. Finally, the weld defect images were presented and compared with a few segmentation methods. After employing the enhanced images, every image quality assessment yields a higher result. Apart from that, in the segmentation stage, the Otsu method obtained the highest average increment, which is 82%. The result performance was evaluated and compared with a few image enhancement techniques. This study produces a number of research contributions, such as developing a new contrast enhancement technique for improving the image quality and improving the segmentation performance. The proposed method is based on a direct technique, which involves a combination of global and local processing.

The main finding can be summarized as follow;

1. A direct enhancement method is developed via statistical information, termed as Hybrid Statistical Enhancement (HSE) method.
2. The image quality is improved, and the segmentation performance has increased using the HSE images.

In spite of the encouraging results obtained, some aspects of the proposed enhancement method can still be considered for improvement. The Hybrid Statistical Enhancement (HSE) method’s performance depends in large part on the threshold information used in the process. The Otsu threshold is used in the present study to supply the threshold information. They are adopted because they are straightforward and simple to use. As a dividing line between the object and the problematic region, the Otsu threshold value is employed. Additionally, the Otsu value is applied alongside the original intensity during the normalization procedure to swap out the problematic intensity. The identified threshold, nevertheless, occasionally has a tendency to yield undesirable outcomes. To enhance the outcomes, perhaps a more effective thresholding technique can be attempted.
